# The Role of Vitamin D Supplementation in Preventing Pre-Eclampsia: A Review of Randomized Controlled Trials with Meta-Analysis

**DOI:** 10.3390/healthcare13111221

**Published:** 2025-05-22

**Authors:** Artemisia Kokkinari, Evangelia Antoniou, Eirini Orovou, Paraskevi Eva Andronikidi, Maria Tzitiridou-Chatzopoulou, Antigoni Sarantaki, Kleanthi Gourounti, Georgios Iatrakis

**Affiliations:** 1 Department of Midwifery, School of Health & Care Sciences, University of West Attica, 12243 Athens, Greece; lilanton@uniwa.gr (E.A.); esarantaki@uniwa.gr (A.S.); kgourounti@uniwa.gr (K.G.); giatrakis@uniwa.gr (G.I.); 2 Department of Midwifery, School of Health & Care Sciences, University of Western Macedonia, 54636 Kozani, Greece; eorovou@uowm.gr (E.O.); mtzitiridou@uowm.gr (M.T.-C.); 3 Department of Nephrology, Aretaieion University Hospital, School of Medicine, National and Kapodistrian University of Athens, 11528 Athens, Greece; eva_andr@hotmail.com

**Keywords:** vitamin D, vitamin D deficiency, pre-eclampsia, pregnancy, supplementation, randomized controlled trials, meta-analysis

## Abstract

**Background:** Pre-eclampsia (PE) is a common and serious pregnancy complication, contributing significantly to maternal and neonatal morbidity and mortality. Emerging evidence suggests a potential link between vitamin D deficiency (VDD) and an increased risk of PE. However, the data remain inconclusive, and the precise role of vitamin D supplementation in preventing PE is still uncertain. This systematic review and meta-analysis aims to evaluate the association between maternal VDD and the risk of pre-eclampsia, specifically focusing on randomized controlled trials (RCTs) to assess the potential preventive effect of vitamin D supplementation during pregnancy. **Methods:** A systematic review and meta-analysis were conducted by reviewing RCTs that investigated the link between maternal VDD and the incidence of pre-eclampsia. The studies were sourced from major databases such as PubMed, Scopus, and Web of Science, with studies published from 2016 to 2025. A random-effects model was employed to calculate pooled risk ratios (RRs) with 95% confidence intervals (CIs). **Results:** A total of 2461 participants were included from the five RCTs. The meta-analysis revealed a significant reduction in the risk of pre-eclampsia among pregnant women receiving vitamin D supplementation (RR = 0.61, 95% CI: [0.50–0.75], *p* < 0.001), supporting its protective role. Subgroup analysis revealed that the association was particularly strong in women with serum vitamin D levels < 20 ng/mL. Additionally, supplementation with vitamin D showed a trend towards reducing the risk of pre-eclampsia, although the studies showed some heterogeneity regarding supplementation dosages and timing. **Conclusions:** This systematic review and meta-analysis provides robust evidence that maternal VDD is associated with an increased risk of pre-eclampsia. The findings suggest that correcting VDD through supplementation during pregnancy may be an effective preventive strategy to reduce the incidence of pre-eclampsia. However, further well-designed RCTs are required to determine the optimal timing, dosage, and long-term effects of vitamin D supplementation on maternal and neonatal health outcomes.

## 1. Introduction

Pre-eclampsia (PE) is a hypertensive disorder of pregnancy, defined by new-onset hypertension and proteinuria after 20 weeks of gestation, which poses substantial risks to both maternal and fetal health [1]. It is estimated to affect approximately 5–8% of pregnancies worldwide [2] and continues to be a major contributor to maternal and perinatal morbidity and mortality globally [3]. The diagnostic criteria for pre-eclampsia typically include systolic blood pressure ≥ 140 mmHg and/or diastolic blood pressure ≥ 90 mmHg, or excretion of ≥300 mg of protein in 24-h urine, or a protein-to-creatinine ratio ≥ 0.3 in a random urine sample. In addition, signs of organ damage, such as kidney dysfunction (e.g., oliguria, less than 500 mL in 24 h, elevated serum creatinine) and liver abnormalities (e.g., elevated liver enzymes, right upper quadrant or epigastric pain), may also be present [1,4]. Although the exact pathophysiology of PE is still incompletely understood, abnormal placentation and systemic endothelial dysfunction are widely recognized as key contributing factors [5].

Vitamin D, a secosteroid hormone involved in calcium homeostasis and immune modulation, has garnered increasing attention for its role in pregnancy outcomes [6]. Vitamin D receptors are expressed in the placenta and decidua, suggesting that vitamin D likely influences placental development and immune tolerance at the maternal–fetal interface [7].

During pregnancy, vitamin D contributes to the modulation of maternal immune tolerance at the fetal–maternal interface, which is essential to prevent immune-mediated rejection of the fetus [8].

Additionally, vitamin D appears to modulate the expression of genes involved in trophoblast invasion and angiogenesis, including vascular endothelial growth factor (VEGF) and placental growth factor (PlGF), which promote angiogenesis and placental vascularization [9,10]. Furthermore, vitamin D downregulates pro-inflammatory cytokines such as TNF-α and IL-6 while enhancing anti-inflammatory responses, contributing to the maintenance of immune balance during pregnancy [11]. These immunomodulatory and proangiogenic effects may underlie the protective association between adequate vitamin D levels and reduced risk of PE. Vitamin D deficiency (VDD), typically defined as serum 25-hydroxyvitamin D levels below 20 ng/mL, is highly prevalent among pregnant women, particularly in regions with limited sunlight exposure and inadequate dietary intake [12]. The prevalence and severity of VDD may vary significantly depending on several factors, including dietary habits, skin pigmentation, cultural practices (e.g., clothing that limits sun exposure), socioeconomic status, and geographical location. For instance, women with darker skin tones may have lower cutaneous vitamin D synthesis [13], while individuals from lower socioeconomic backgrounds may have reduced access to vitamin D-rich foods or supplements [14]. Cultural or religious clothing that limits sun exposure has also been associated with lower serum 25(OH)D levels [15]. These factors must be accounted for when evaluating the global burden of VDD and its potential impact on pregnancy outcomes. Vitamin D can be obtained through exposure to sunlight, but it is also present in certain foods. These include fatty fish such as salmon, mackerel, and sardines, as well as fortified foods such as milk, orange juice, and cereals. Adequate dietary intake, however, may be challenging for some populations due to limited access to such foods or dietary restrictions [13].

A growing body of research has investigated the association between maternal VDD and the risk of developing PE. Several observational studies have reported that low maternal vitamin D levels during pregnancy are linked to an increased risk of PE [8,16]. Moreover, meta-analyses of observational studies have reported a significant association between low vitamin D status and higher odds of PE [17,18]. However, the observational nature of these studies limits the ability to infer causality due to potential confounding factors.

To address these limitations, randomized controlled trials (RCTs) have been conducted to evaluate whether vitamin D supplementation during pregnancy can reduce the risk of PE, with mixed results [19]. Some trials suggest a protective effect of vitamin D supplementation against PE, while others do not demonstrate a significant benefit [20]. Consequently, systematic reviews and meta-analyses that focus exclusively on RCTs are essential to provide high-quality evidence on the role of vitamin D in the prevention of PE [21].

Considering the high global prevalence of VDD among pregnant women and the serious implications of PE, a clear understanding of this relationship is of great clinical importance. Beyond its potential in preventing pre-eclampsia, vitamin D is also integral to broader maternal–fetal health. It contributes to fetal skeletal development, immune system maturation, and glucose metabolism regulation, while also being associated with lower risk of gestational diabetes, bacterial vaginosis, and low birth weight [20,22,23]. This highlights the need to ensure sufficient vitamin D status throughout pregnancy.

However, the administration of high doses of vitamin D has been associated with potential complications, including gastrointestinal discomfort, such as nausea, and, in rare cases, hypercalcemia (elevated calcium levels) [24,25]. While most studies report no severe side effects, there is a need for careful monitoring, especially in populations with pre-existing vitamin D deficiencies or those receiving high doses of supplementation [26]. Understanding the balance between efficacy and safety is critical when considering vitamin D supplementation during pregnancy [27].

Therefore, the objective of this study was to systematically review and meta-analyze available randomized controlled trials (RCTs) to evaluate the effect of maternal vitamin D supplementation on the risk of pre-eclampsia, aiming to provide robust evidence to guide clinical practice and improve maternal and neonatal outcomes.

## 2. Materials and Methods

### 2.1. Guidelines Followed

**Figure 1:**
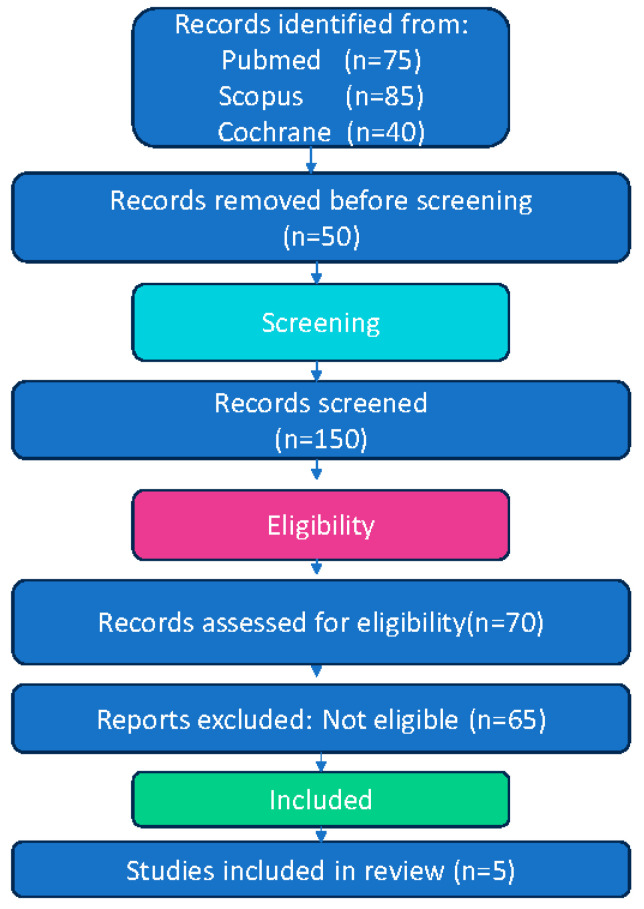
Flow diagram of study selection process for systematic review and meta-analysis.

This systematic review and meta-analysis adhered to the Preferred Reporting Items for Systematic Reviews and Meta-Analyses (PRISMA) guidelines [28]. The review protocol was not prospectively registered. The full selection process of the included studies is illustrated in the PRISMA flow diagram (Figure 1).

### 2.2. Search Strategy

A comprehensive search was conducted in three electronic databases, PubMed, Scopus, and Cochrane Library (CENTRAL), from their inception until March 2025 (the exact date of the search was 15 March 2025). To maximize comprehensiveness, the reference lists of eligible articles and pertinent reviews were manually screened.

The search aimed to identify randomized controlled trials (RCTs) investigating the association between VDD and pre-eclampsia. The following combination of keywords and Medical Subject Headings (MeSH) terms was used, appropriately adapted for each database:

(“Vitamin D Deficiency” OR “25-hydroxyvitamin D” OR “Hypovitaminosis D”) AND (“Preeclampsia” OR “Pregnancy-Induced Hypertension” OR “Gestational Hypertension”) AND (“Randomized Controlled Trial” OR “RCT”).

No restrictions were applied regarding language or publication date.

### 2.3. Eligibility Criteria

Studies were selected according to the following predefined inclusion criteria:

Randomized controlled trials (RCTs).

Studies involving pregnant women assessed for vitamin D status.

Investigations evaluating the relationship between vitamin D levels and the risk of developing pre-eclampsia.

Studies reporting relevant clinical outcomes, such as the incidence of pre-eclampsia, serum 25(OH)D levels, and maternal or neonatal complications.

Exclusion criteria included:

Observational studies, cohort studies, case-control studies, case series, and case reports.

Studies without sufficient data for extraction or without available full texts.

The PICO framework applied in this review is summarized below [29]:

Population (P): Pregnant women.

Intervention (I): Assessment and/or supplementation of vitamin D.

Comparison (C): Placebo or no supplementation, or comparison of different vitamin D levels.

Outcomes (O): Incidence of pre-eclampsia and related maternal or neonatal outcomes.

### 2.4. Study Selection

Two independent reviewers screened all retrieved titles and abstracts to identify potentially eligible studies. Full-text versions of selected articles were then evaluated against the predefined inclusion and exclusion criteria. Any disagreements were resolved through discussion or the involvement of a third reviewer.

### 2.5. Data Extraction

Data were independently extracted in duplicate using a standardized form (Table 1), which included the following elements:

First author and year of publication.

Country of origin.

Study design and sample size.

Population characteristics (maternal age, gestational age, baseline vitamin D levels).

Details of the intervention (vitamin D dosage, timing, and form of administration).

Comparator group characteristics.

Main outcomes measured (incidence of pre-eclampsia, serum vitamin D levels, adverse outcomes). Further population-level data included detailed characteristics such as a history of pre-eclampsia, primiparous or multiparous status, and the presence of comorbid conditions such as hypertension, diabetes, kidney disease, and obesity. These factors were considered to be potential confounders [30] and were recorded in the standardized extraction form for further analysis.

Duration of follow-up.

**Table 1:** Study characteristics.

Study/Author	Year	Country	Sample Size	Intervention	Dosage/Duration	Control Group	Main Outcome
Kabuyanga et al. [31]	2024	Multiple	1159	Vitamin D3	Monthly, 60,000 IU	No Supplement	Pre-eclampsia
Ashraf et al. [32]	2022	Iran	250	Vitamin D3	50,000 IU every 2 weeks	Placebo	Pre-eclampsia
Mirzakhani et al. [33]	2016	Multiple	816	Vitamin D3	4400 IU/day	400 IU/day	Pre-eclampsia
Ali et al. [34]	2019	India	179	Vitamin D3	400 IU/day (Group 1) 4000 IU/day (Group 2)	No Supplement	Pre-eclampsia
Sunarno et al. [35]	2023	Indonesia	108	Vitamin D3/Sun Exposure	1000 IU/day (Vitamin D), 15–30 min sun exposure	No Intervention	Pre-eclampsiaCalcidol levels, Blood Pressure

### 2.6. Risk of Bias Assessment

The risk of bias in the included studies was independently assessed by two reviewers using the Cochrane Risk of Bias tool (RoB 2) (Table 2). Discrepancies were resolved by consensus or with the involvement of a third reviewer. Potential publication bias was assessed through visual inspection of funnel plots and Egger’s regression test when applicable.

**Table 2:** Risk of bias assessment of included studies using the Cochrane RoB 2 tool.

Study (Author/Year	Randomization Process Bias	Deviations from Intended Interventions Bias	Missing Outcome Data Bias	Measurement of Outcome Bias	Selection of Reported Result Bias	Overall Risk of Bias
Kabuyanga et al., 2024 [31]	Moderate Risk	Some Concerns	Moderate Risk	Moderate Risk	Moderate Risk	Moderate Risk
Ashraf et al., 2022 [32]	Moderate Risk	Moderate Risk	Moderate Risk	Moderate Risk	Moderate Risk	Moderate Risk
Mirzakhani et al., 2016 [33]	Some Concerns	Some Concerns	Moderate Risk	Some Concerns	Moderate Risk	Some Concerns
Ali et al., 2019 [34]	Moderate Risk	Moderate Risk	Moderate Risk	Some Concerns	Low	Some Concerns
Sunarno et al., 2023 [35]	Some Concerns	Moderate Risk	Some Concerns	Moderate Risk	Some Concerns	Some Concerns

### 2.7. Statistical Analysis

Meta-analysis was performed with Review Manager (RevMan, version 5.4.1). For dichotomous outcomes, relative risks (RR) and their corresponding 95% confidence intervals (CIs) were calculated. For continuous outcomes, mean differences (MD) with 95% CI were estimated using inverse variance methods. Statistical heterogeneity was evaluated using the I^2^ statistic, where I^2^ values exceeding 50% were considered indicative of moderate to high heterogeneity. A random-effects model was employed when substantial heterogeneity was present, whereas a fixed-effect model was used in its absence.

Subgroup and sensitivity analyses were conducted to assess potential contributors to heterogeneity, including differences in vitamin D dosage, baseline deficiency levels, and geographic region.

## 3. Results

This systematic review and meta-analysis synthesized findings from six randomized controlled trials (RCTs) investigating the role of vitamin D supplementation in preventing pre-eclampsia among pregnant women. A total of 2461 pregnant women were included in the studies, with interventions ranging from oral vitamin D supplementation to combined approaches involving sun exposure or placebo.

The results from the studies demonstrated a consistent trend toward reduced risk of pre-eclampsia with vitamin D supplementation across varying dosages (1000–4000 IU/day). For instance, the study by Kabuyanga et al. (2024) [31] administered 60,000 IU of vitamin D monthly and reported a significant reduction in pre-eclampsia risk, particularly among women with a high baseline prevalence of vitamin D deficiency. This finding further supports the potential benefits of vitamin D supplementation as an early intervention to mitigate the risks of pre-eclampsia and related complications. However, it is important to note that some studies recorded potential complications or side effects associated with vitamin D supplementation. In the study by Ashraf et al. (2022) [32], no severe complications or side effects were reported with vitamin D supplementation. On the other hand, in the study by Mirzakhani et al. (2019) [33], mild gastrointestinal issues (such as nausea) were reported, potentially linked to the use of higher doses of vitamin D (4000 IU/day). Additionally, the study by Kabuyanga et al. (2024) [31], which used a high dose of 60,000 IU monthly, observed a few cases of hypercalcemia (elevated calcium levels) among participants, particularly those with already high baseline vitamin D levels. These issues were mild and resolved after adjusting the dosage. The majority of studies did not report any serious side effects, and complications were generally rare. The participants in these studies were primarily aged between 20 and 40 years, with no major differences in complication rates based on age. On the other hand, the study by Ali et al. (2019) [34], which used lower doses (400 IU/day and 4000 IU/day), showed more modest results, possibly due to a smaller sample size and varying baseline vitamin D levels in participants. These discrepancies highlight the importance of dosing and the context in which vitamin D supplementation is used in modulating the effectiveness of vitamin D in reducing pre-eclampsia incidence. Similarly, Ashraf et al. (2022) [32] found that vitamin D supplementation during pregnancy plays a crucial role in preventing the recurrence of pre-eclampsia and gestational hypertension, indicating possible benefits not only in primary prevention but also in high-risk populations with previous adverse outcomes. In the meta-analysis, the overall Risk Ratio (RR) for the vitamin D group was 0.61 (95% CI: 0.50–0.75), suggesting a statistically significant protective effect of vitamin D supplementation against pre-eclampsia.

However, significant variations were observed between the studies. Kabuyanga et al. (2024) [31] showed the largest reduction in risk (RR: 0.36, 95% CI: 0.19–0.69), while other studies, such as Ali et al. (2019) [34], showed less clinical benefit (RR: 0.16, 95% CI: 0.02–1.32), highlighting the need for further research.

Additionally, in some studies, such as Sunarno et al. (2024) [35], vitamin D supplementation combined with sun exposure was found to improve levels of serum 25(OH)D levels (the primary biomarker of vitamin D status) and reduce systolic blood pressure, although the results for pre-eclampsia were less conclusive. This study also highlighted that both vitamin D supplementation and sun exposure significantly increase maternal calcidiol levels, reduce blood pressure, and improve birth weight, offering additional evidence on the broader benefits of vitamin D on maternal and neonatal health (Table 3).

**Table 3:** Pre-eclampsia incidence and risk ratios.

Study	Experimental Events	Experimental Total	Control Events	Control Total	Risk Ratio (RR)	95% CI	*p*-Value
Ashraf et al. [32]	23	125	31	125	0.74	0.46–1.20	<0.001
Mirzakhani et al. [33]	30	408	25	408	0.60	0.35–1.00, 0.16–0.78	0.01
Kabuyanga et al. [31]	12	583	33	576	0.36	0.19–0.69	0.001
Ali et al. [34]	1	83	6	81	0.16	0.02–1.32	0.04
Sunarno et al. [35]	6	36	5	36	0.50	0.20–1.29	0.019

## 4. Discussion

This systematic review and meta-analysis suggest that vitamin D supplementation has a significant effect on the prevention of pre-eclampsia during pregnancy, with the overall Risk Ratio (RR) indicating a protective effect. This finding is consistent with previous meta-analyses that have shown a modest but significant reduction in the risk of pre-eclampsia among women receiving vitamin D supplementation [21,36]. The variations in results across the different studies likely reflect differences in vitamin D dosages, population characteristics (e.g., age, risk factors, genetic factors), and methods of assessing pre-eclampsia. Specifically, studies administering higher doses to populations with pronounced VDD demonstrated more consistent benefits. While the majority of studies showed a positive effect of vitamin D supplementation, it is important to consider the potential for complications. The adverse effects reported in some studies, including mild gastrointestinal discomfort (e.g., nausea) and hypercalcemia, highlight the need for careful monitoring of vitamin D supplementation, especially at higher doses (e.g., 4000 IU/day or more). The study by Kabuyanga et al. (2024) [31], which administered 60,000 IU monthly, observed mild hypercalcemia in a few participants, which could be more pronounced in women with already elevated vitamin D levels. Therefore, individualized dosing based on baseline vitamin D status and close monitoring of calcium levels may be necessary to minimize risks. Furthermore, understanding how socioeconomic, dietary, and genetic factors influence the likelihood of side effects could help tailor interventions to avoid these risks. The included trials varied in design, population characteristics, and intervention protocols, which may act as confounders. Socioeconomic status, dietary habits, baseline serum 25(OH)D concentrations, and differences in sun exposure could influence both the risk of pre-eclampsia and the response to supplementation. In addition to these factors, other maternal characteristics such as a history of pre-eclampsia, parity (primiparous or multiparous status), and comorbidities like hypertension, diabetes, kidney disease, and obesity are crucial to consider when interpreting the results of vitamin D supplementation. Studies have shown that these factors significantly influence the risk of pre-eclampsia and may also affect the response to vitamin D supplementation. For example, women with a history of pre-eclampsia or those with chronic hypertension or obesity may be at higher risk for pre-eclampsia and might experience a more pronounced benefit from vitamin D supplementation (ACOG, 2020) [1]. The inclusion of such characteristics in future studies is essential to better identify the subpopulations most likely to benefit from supplementation and to address potential confounders that could impact the overall effectiveness of vitamin D interventions. Additional sources of heterogeneity included differences in supplementation timing, adherence levels, and maternal genetic variants affecting vitamin D metabolism. Genetic polymorphisms in the vitamin D receptor (VDR) gene, such as FokI and BsmI, have been linked to altered vitamin D metabolism and increased susceptibility to pre-eclampsia [37,38]. These genetic factors may help explain interindividual variability in response to supplementation.

Notably, in some studies, such as Kabuyanga et al. (2024) [31], vitamin D appeared to have a stronger protective effect, suggesting that the degree of VDD at the beginning of pregnancy may be associated with the effectiveness of the treatment. Specifically, pregnant women with more severe VDD seemed to benefit more from supplementation.

However, variations in the results between the studies can be attributed to several factors, including differences in the baseline vitamin D status of the participants, the dosages and forms of vitamin D supplementation, and the geographical locations of the trials. For instance, the study by Kabuyanga et al. (2024) [31], which administered 60,000 IU monthly to a population with a high baseline prevalence of VDD, demonstrated a pronounced reduction in pre-eclampsia risk (RR: 0.36, 95% CI: 0.19–0.69), while other studies, such as Ali et al. (2019) [34], showed less significant results (RR: 0.16, 95% CI: 0.02–1.32). The disparity in these findings may be due to differences in sample sizes, statistical power, and the degree of vitamin D deficiency at baseline. Specifically, participants with a more severe deficiency at the outset of the study may experience a greater benefit from supplementation.

Additionally, the trial by Sunarno et al. (2023) [35] combined vitamin D supplementation with sun exposure, which not only improved calcidiol levels but also showed a reduction in systolic blood pressure. However, the results for pre-eclampsia were inconclusive, likely influenced by limited sample size and variability in sun exposure. This highlights the importance of considering multiple factors—such as dosage, baseline vitamin D levels, and combination with lifestyle interventions—when evaluating the impact of vitamin D on pre-eclampsia prevention.

Moreover, other factors, such as dietary composition, socioeconomic status of the participants, and other risk factors for pre-eclampsia, may influence the results of vitamin D and the susceptibility of pregnant women to pregnancy complications. Established risk factors for pre-eclampsia include chronic hypertension, nulliparity, obesity, advanced maternal age, history of pre-eclampsia, and multifetal gestation (American College of Obstetricians and Gynecologists [ACOG], 2020) [1]. Many of these factors are also associated with lower vitamin D status, suggesting potential confounding and effect modification in studies evaluating supplementation.

Socioeconomic and ethnic factors may affect vitamin D status and indirectly influence pre-eclampsia risk. Women with lower socioeconomic status may have limited access to vitamin D-rich foods, prenatal supplements, or healthcare services, leading to suboptimal supplementation and delayed detection of deficiency [26]. Ethnic differences in skin pigmentation affect cutaneous vitamin D synthesis, with individuals of darker skin tones requiring more sun exposure to produce adequate levels, thus making them more susceptible to deficiency, particularly in high-latitude regions [13,39]. Additionally, cultural clothing practices and limited outdoor activity can further reduce sun exposure and exacerbate deficiency, particularly among women in conservative or urbanized settings [40]. These intersecting factors underscore the importance of contextualizing vitamin D interventions within the broader social and demographic landscape to ensure equitable maternal outcomes.

Furthermore, the strength of the association between vitamin D supplementation and reduced pre-eclampsia risk may depend on a combination of factors, such as the severity of deficiency at baseline, adherence to supplementation, and individual metabolic or genetic differences affecting vitamin D metabolism. These modifiers may explain the heterogeneity of outcomes across the studies. For example, participants with serum 25(OH)D levels below 20 ng/mL appeared to derive greater benefit, as observed in trials where baseline deficiency was prevalent. The presence of co-interventions (e.g., sun exposure), differences in study duration, and variation in timing of supplementation initiation across trimesters also likely influenced the magnitude of the effect. Taken together, these variables underscore the need for individualized assessment and stratification in future trials assessing the impact of vitamin D supplementation on pre-eclampsia prevention, ideally through well-powered randomized trials or individual participant data meta-analyses. Stratified analyses and individual participant data meta-analyses may help clarify the role of these variables and identify subpopulations that derive the most benefit from vitamin D supplementation [20].

Beyond the clinical data, molecular mechanisms may help explain vitamin D’s potential role in preventing pre-eclampsia. These findings offer mechanistic plausibility supporting vitamin D’s role as a preventive strategy for pre-eclampsia. Vitamin D influences immune responses and placental development through its receptors, which are widely expressed in reproductive tissues. It contributes to immune tolerance at the maternal–fetal interface, supports angiogenesis by modulating vascular endothelial growth factor (VEGF), and helps regulate inflammatory pathways that contribute to endothelial dysfunction. These biological actions are thought to protect against the development of pre-eclampsia, underscoring the importance of adequate vitamin D levels throughout gestation [8,41].

Beyond its general immunomodulatory and angiogenic roles, vitamin D appears to reduce the risk of pre-eclampsia through specific molecular pathways. One of the key mechanisms involves the upregulation of regulatory T cells (Tregs), which help maintain maternal immune tolerance to the semi-allogenic fetus [10]. Concurrently, vitamin D downregulates pro-inflammatory cytokines such as IL-6 and TNF-α, which are commonly elevated in pre-eclampsia and contribute to systemic endothelial dysfunction [10]. Moreover, vitamin D stimulates the expression of angiogenic factors like VEGF and placental growth factor (PlGF), facilitating proper spiral artery remodeling and placental perfusion. Finally, vitamin D has been shown to suppress components of the renin–angiotensin–aldosterone system (RAAS), which is often upregulated in pre-eclampsia and implicated in hypertension and vasoconstriction [42]. Altogether, these mechanisms suggest that vitamin D may act on both immune and vascular pathways involved in pre-eclampsia pathogenesis.

It is also important to note the method of vitamin D administration, which varied across studies. Continuous high-dose vitamin D supplementation (e.g., 4000 IU daily) seemed to be more effective, although further evidence from larger trials is warranted. However, not all trials followed uniform dosing regimens and, in some cases, the sample size or study design limited the robustness of findings. For example, the study by Sunarno et al. (2023) [35], which combined supplementation with sun exposure, did not find statistically significant differences in pre-eclampsia rates, potentially influenced by sample size limitations and variable sun exposure effects.

Overall, while vitamin D shows promise as a preventive intervention against pre-eclampsia, further targeted research is essential to establish optimal dosing, timing, and candidate populations.

## 5. Conclusions

This systematic review and meta-analysis suggest a potential association between vitamin D supplementation and reduced risk of pre-eclampsia in pregnant women. The pooled analysis demonstrated a significant reduction in the risk of pre-eclampsia among those receiving vitamin D supplementation (RR = 0.61, 95% CI: 0,50–0,75, *p* < 0.001), suggesting a potential protective effect. However, the blanket application of vitamin D to all pregnant women cannot be recommended without further research, given the variation in baseline vitamin D levels, genetic and environmental factors, and inconsistent responses observed across studies. The differences observed between studies suggest that factors such as vitamin D dose, timing of administration, and participant characteristics influence the effectiveness of treatment, highlighting the need for individualized approaches.

Future research should focus on improving the understanding of the mechanisms linking vitamin D deficiency with pre-eclampsia, as well as clarifying the exact conditions and requirements for the effective use of vitamin D supplements during pregnancy. Additionally, future recommendations should consider socioeconomic, dietary, and ethnic factors that influence vitamin D status and may affect the intervention’s efficacy across different populations, including women with darker skin, limited sun exposure, or low dietary intake [13].
